# Factors Associated with Nursing Student Satisfaction with Their Clinical Learning Environment at Wolkite University in Southwest Ethiopia

**DOI:** 10.1155/2022/3465651

**Published:** 2022-10-25

**Authors:** Ayana Benti Terefe, Tolesa Gemeda Gudeta

**Affiliations:** Department of Nursing, College of Medicine and Health Science, Wolkite University, Wolkite, Ethiopia

## Abstract

**Background:**

The clinical learning environment has been defined as “an interactive network of strengths within the clinical environment that influence the clinical learning outcomes of students. Understanding students' level of satisfaction with their clinical learning environment is crucial to ensuring the required teaching and learning process. Therefore, this study was designed to assess the level of satisfaction with the CLE among nursing students at the Wolkite University of Ethiopia.

**Methods:**

A cross-sectional study was conducted at Wolkite University. This study was carried out in March 2022 on 208 student nurses selected by purposive and convenient sampling techniques. This study used the English version of the Clinical Learning Environment and Supervision + Nurse Teacher (CLES + *T*) evaluation scale. The collected data were cleaned, coded, and entered into Epi data 3.1, and then, statistical analyses were performed using SPSS version 26. Bivariate and multivariable logistic regression models were used to identify factors associated with the satisfaction level of nursing students towards CLE. Adjusted odds ratios (AORs) with 95% confidence intervals (CIs) were calculated, and *p* values < 0.05 were considered to indicate statistical significance.

**Results:**

More than half of the study participants were females, and nearly half (50.5%) of the participants were in year three of the nursing program. The study found that 39.9% (95%CI: 32.2%, 46.6%) of students were satisfied with their CLE. Factors that had a significant association with the satisfaction level of nursing students towards CLE were gender (female students) (AOR = 16.053 (6.397, 40.286)), year of study (4th year) (AOR = 6.296 (2.679, 14.796)), and the type of the hospital in which their last clinical placement was held (at a primary hospital) (AOR = 2.961 (1.122, 7.815)).

**Conclusion:**

Effective nursing education programs need to be developed to increase satisfaction with clinical practice and to promote positive emotional regulation in nursing students. Nurse practitioners and managers should be aware of their important role in the professional development of students and their satisfaction with clinical placements.

## 1. Background

Nursing education should include instruction and practice in a clinical learning setting. A clinical learning environment (CLE) is an environment where clinical skills are acquired by direct or indirect supervision by preceptors and nurse teachers that are both facilitators and responsible for the student's learning in clinical settings. In nursing, the CLE is an essential part of developing skills and integrating theoretical knowledge, clinical practice, and professionalism [1–4].

CLE is a crucial component of the learning process in nursing education. Learning in the clinical practice environment is an essential and core component of nursing education because nursing is a skill-based profession. CLE plays a crucial role in enabling students to develop their professional skills and competencies as they pursue becoming registered nurses [5–7]. To prepare nursing students for the rigors of professional practice, learning in the practice setting is crucial. Clinical practice involves training in a safe and secure environment, without the pressure of real-world performance, for practicing skills; it constitutes a bridge between academic learning and the clinical setting in which students integrate theory and practice and develop a reflective stance [8–11].

Clinical education facilitates the integration and translation of nursing students' theoretical knowledge from academia to practice, thereby enabling learning through learning in a realistic clinical setting [12]. For nurses to effectively perform the myriads of tasks, it depends on their capacity to apply theory to practice. An effective learning process in the clinical setting is essential to combine cognitive, psychomotor, and appropriate attitudes in such a way to benefit both the learning needs of the student and the nursing care needs of the client [2, 13–15].

Student satisfaction with clinical experience and the overall simulated learning environment was a good indicator of the quality of nursing education. Student satisfaction is an important element of the study of the effectiveness of a clinical-based learning environment. Student satisfaction is crucial for meaningful and engaged learning because it encourages active, purposeful engagement in clinical learning experiences [16–18].

The CLE is complicated and dynamic. Many variables, including the kind of supervisor, the standard of student feedback, the setting, and the participants, can affect the CLE. Studies have revealed that challenges faced by nursing students during their CLE have an impact on their general health and interfere with their learning process [19, 20].

Numerous variables, including social, psychological, environmental, and academic factors, can have an impact on a nursing student's satisfaction with their clinical experience and their subsequent involvement in the process [21, 22]. The preference for the practice center, the mean student grade, the distance to the practice center, the number of students assigned to the clinical educator, the type of service, the type of center, and the type of management were the factors influencing satisfaction with their CLE [1, 18, 23].

Many researchers explored the different factors that contributed to the successful development of competencies over the course of clinical placement. The followings are the main learning domains affecting student nurses, ward atmosphere (WA), the leadership of the ward manager (WM), and a supervisory relationship with a nurse teacher (NT) [1, 3, 15, 17, 24–28].

Although nursing students' satisfaction with their CLE is necessary, studies conducted in different parts of the world found that an unconstructive CLE can hinder the attainment of learning targets and delay the gaining of skills, attrition, and dissatisfaction among nursing students. Research conducted in many countries indicates that negative experiences with CLE hinder the achievement of learning outcomes, exacerbating an international nursing shortage [20, 29–31].

Even though a careful understanding of students' satisfaction with their CLE is essential for securing the required teaching and learning process, there is a scarcity of research related to nursing students towards CLE satisfaction in Ethiopia. The research findings will contribute to the limited body of knowledge regarding the topic. The need to improve clinical nursing education is an important aspect of the training of nurses. Therefore, this study forwarded ways of clinical teaching improvement and will enhance the way department heads and nurse educators assume their primary role of improving nursing students during their clinical practice. In addition to nursing education, the results of the study will also be helpful for nursing practice, administration, and further research. Thus, this study was designed to assess the level of satisfaction with the CLE among nursing students at Wolkite University in Ethiopia.

## 2. Methods

### 2.1. Study Setting

This study was conducted at Wolkite University, SNNP, Ethiopia. Wolkite University is one of the 3^rd^ generation federal universities in Ethiopia. Wolkite University (WKU) is currently located at SNNPR, Gurage Zone, 170 km south-west of Addis Ababa on the way to Jimma. Currently, the university offers undergraduate, postgraduate, and Ph.D. programs.

### 2.2. Study Design and Period

A cross-sectional study using a quantitative research design was conducted on March 10–15 2022.

### 2.3. Population and Eligibility Criteria

All third and fourth-year students were included to participate in the study. The reason behind including those students is that they have already started their clinical attachments with different courses. The inclusion criteria in this research were the students studying in the third and fourth years in the nursing department, consent to participate in the study, and filling out the questionnaire. The exclusion criteria included unwillingness to participate in the study or absence due to educational leave at the time of the study. The data were collected from all the studied populations.

### 2.4. Sampling Technique

Purposive and convenient sampling techniques were implemented. It is purposive as only third and fourth-year students have been selected to take part in the study. It was convenient because students of those year levels who were available and interested in participating were selected.

### 2.5. Variables of the Study

#### 2.5.1. Dependent Variable

Satisfaction of nursing students with CLE is a dependent variable in this study.

#### 2.5.2. Independent Variable

- *Demographic variables are as follows*: gender, year of study, type of the nursing ward of the last clinical placement, type of the hospital in which clinical placement was held, and number of meetings with nurse teachers during the latest clinical placement.

### 2.6. Operational Definition

**Satisfaction with CLE:** The Clinical Learning Environment, Supervision, and Teacher (CLES + *T*) scale was used to operationalize satisfaction with CLE. In this case, it was operationalized as follows: students are satisfied if they get more than the mean value of the total satisfaction score and vice versa.

### 2.7. The Data Collection Tool and Procedure

Self-administered questionnaires were used. In this study, the CLES + *T* scale was used. In addition to that, questionnaire on demographic data including the gender, year of study, type of nursing ward of last clinical placement, type of the hospital in which clinical placement was held, and frequency of meeting with nurse teachers during the latest clinical placement was used. We used the English version of the CLES + *T* assessment scale. This scale of psychometric tests includes a total of 34 elements in five subdimensions [24]. The subdimensions are as follows: pedagogical atmosphere of the ward (nine items), leadership style of the ward manager (four items), premises of nursing of the ward (four items), supervisory relationships (eight items), and the nurse teacher's role in clinical practice (nine items). A Likert scale of 1 to 5 points was used to grade the questions. The scores were as follows: 1 = strongly disagree, 2 = disagree, 3 = neutral, 4 = agree, and 5 = strongly agree. Each student's questionnaire's total mean score was determined by adding up the mean scores of every question. Each student's scores on the questions that make up each of the five subdimensions were also calculated. Higher ratings represent greater agreement with the statements. By averaging the students' overall satisfaction scores, the degree of their contentment with their clinical learning environment was ascertained. The data were collected by two MSc midwifery teachers and one supervisor.

### 2.8. Statistical Analysis

The collected data were cleaned, coded, and entered into Epi data 3.1, and then, statistical analyses were performed using SPSS version 26. Students' demographic features are displayed as frequencies and percentages. The variables from the bivariate analysis that had a *p* value of less than 0.25 were added to multivariable logistic regression models to help control confounding variables. To explain the relative impact of independent variables on satisfaction with the clinical learning environment, multivariable logistic regression analysis with a backward stepwise approach of variable selection was utilized. The strength of the link between numerous independent variables and outcome variables was assessed using the adjusted odds ratio after confounding variables were taken into account. The Hosmer– Lemeshow assumption test was used to evaluate the model's goodness of fitness and found that it was properly fitted. Multivariable logistic regression finally reached statistical significance at a *p* value of < 0.05.

### 2.9. Data Quality Management

Before actual data collection, a pretest was conducted on 5% [10] of the sample of randomly selected nursing students at a nearby university by data collectors. The reliability of the data collection tool was measured (Cronbach's alpha of the overall items was 0.92), data collection time was estimated, and some modifications such as logical order and rewriting items difficult to understand were made as well. At the end of each day of data collection, the principal investigator and supervisor reviewed the questionnaire to ensure its consistency and completeness.

## 3. Results

Regarding the sample's demographics data, 47.6% were males and 52.4% were females, with ages ranging from 18 to 34 years, with a mean of 21.08 years and a standard deviation of 2.23 years. Nearly half of the participants were in year three of the nursing program (50.5%). The majority of participants (44.7%) attended the medical ward in their last clinical placement. Of the participants, 55.7% attended a specialized care center for the last clinical learning site and 38.9% met a nurse teacher frequently during the latest clinical placement (Table 1).

### 3.1. Satisfaction Level of Nursing Students towards CLE

The minimum and maximum scores were 34 and 170, respectively. The mean score for total satisfaction towards CLE among nursing students after adding all items was 3.07 (SD = 0.59). The total score of the respondents who have high satisfaction was 39.9%(95%CI: 32.2%, 46.6%) (*n* = 83), and the total score of those having low satisfaction was 60.1% (*n* = 125).  Table 2 shows the level of satisfaction towards CLE among nursing students at Wolkite University. There were five dimensions of satisfaction towards CLE, and the pedagogical atmosphere (50.0%) was the highest, whereas the role of the nurse teacher (34.6%) was the lowest (Table 2).

## 4. Associated Factors with the Satisfaction Level of Nursing Students towards CLE

Both bivariate and multivariable logistic regression analyses were carried out to identify factors associated with the satisfaction level of nursing students towards CLE. Accordingly, in bivariate analyses, gender, age, year of study, type of the hospital in which their last clinical placement was held, and frequency of their meeting with the nurse teacher during their latest clinical placement were significantly associated with a satisfaction level of nursing students towards CLE at *p* value < 0.25.

All independent variables with *p* < 0.25 in the bivariate logistic regression analysis were entered into the multivariable logistic regression analysis to identify the final factors associated with the satisfaction level of nursing students towards CLE. Backward logistic regression was used for selecting variables in the final model. In the multivariable logistic regression analysis, gender, year of study, and type of the hospital in which their last clinical placement was held were factors associated with the satisfaction level of nursing students towards CLE.

Female students were about 16.053 (AOR = 16.053 (6.397, 40.286)) more likely to be satisfied with CLE than male students. Those students who were in the fourth year of their study were about 6.296 times (AOR = 6.296 (2.679, 14.796)) more likely to be satisfied with CLE than students in the third year. Additionally, students who attended their last clinical placement in a primary hospital were about 2.961 times (AOR = 2.961 (1.122, 7.815)) more likely to be satisfied with CLE than students who were attending their last clinical placement at a specialized care center (Table 3).

## 5. Discussion

Our study aimed to assess satisfaction levels towards CLE and its associated factors among selected nursing students at Wolkite University. Accordingly, the magnitude of students' satisfaction with CLE was 39.9% (95%CI: 32.2%, 46.6%). The finding in this study is similar to that reported by a study conducted in Rwanda, in which (40%) of the participants were satisfied [28]. On the other hand, the magnitude of students satisfied with CLE in this study was lower than that in studies conducted in Nepal (88%) [3], at King Saud University [4], the three universities in Cyprus [17], in the Universiti Kebangsaan Malaysia [25], and in India (54.86%) [27]. These discrepancies may be due to differences in the time of research conducted and the difference in the study settings. Furthermore, the discrepancy between the numbers of the sample size used can also affect the difference in the satisfaction level.

The present study' findings showed that the most satisfactory area for student nurses was the pedagogical atmosphere (50%) in their clinical learning environment and that they were least satisfied with the role of the nurse teacher (34.6) and nursing care of the ward (35.1) in their clinical learning environment. Contrary to this, the most satisfactory area for student nurses was the leadership style (1.44) of their mentors in their clinical learning environment, and they were least satisfied with the student-nurse relationship (1.41) and content context balance (1.41) in their clinical learning environment [27]. Furthermore, a study conducted in Ghana demonstrated that the most satisfactory area for student nurses was the leadership style of the ward manager (3.63) [1]. The differences in mean scores in dimensions of the CLES + *T* were found in different studies that we reviewed. This might be due to different studies considering nursing students in different year levels.

The gender of students was associated with the satisfaction level of nursing students towards CLE. The finding of the present study was supported by a qualitative study that was conducted on nursing students' challenges in a CLE at the school of nursing and midwifery at Addis Ababa University. The result showed that lack of interest is different among gender (male vs. female) as male feel more undermined to study nursing [19]. This might be because the majority of participants were females. On the contrary, Karim et al. [25] found that there was no significant relationship between gender and the level of satisfaction.

In addition, the year of study was strongly associated with the satisfaction level of nursing students towards CLE. The finding of the present study was supported by a study that was conducted on the perception of the CLE among nursing students in Nepal. The result showed that the satisfaction of students towards CLE is more as their year of academic study increases [3]. This finding is also confirmed by a different study performed previously [11, 17, 22]. The reason behind this may be due that their satisfaction increased as students progressed through the program that the higher-year students expressed higher confidence in clinical knowledge and skills. On the contrary, a study conducted in Ghana found that the mean CLES + *T* score was not associated with the year of study even though third-year students had higher scores than fourth-year students [1].

Our findings highlight that the satisfaction with a CLE of nursing students is associated with the type of hospital in which their last clinical placement was held. In this case, students who attended their last clinical placement in a primary hospital were more satisfied with their CLE. This is also supported by another study [10]. The possible justification may be due to the attitudes of the nursing staff and the strong support of students by the staff in primary hospitals being high compared to in specialized care centers. On the other hand, the relationship between the last clinical placement in the previous semester and the level of satisfaction was not significant in another study [25].

## 6. Conclusion

In conclusion, efficient nursing education programs must be created to improve clinical practice satisfaction and support good emotional regulation in nursing students. Nursing teachers and managers should focus on how to increase the satisfaction level of students. The importance of their contributions to student's professional growth and satisfaction with clinical placements should be understood by nurse educators and department leaders. Gender, year of study, and type of the hospital in which their last clinical placement was held were factors associated with the satisfaction level of nursing students towards CLE. Improving the positive perception of male students towards CLE and improving nursing students' satisfaction starting from the time they started clinical practice will be expected from all responsible bodies. Furthermore, making a close relationship between the nursing department which provides theory and hospitals in which students attend their clinical practice will be recommended to increase the satisfaction level of nursing students towards CLE.

## Figures and Tables

**Table 1:** Distribution of the study participants across demographics (*n* = 208).

Variables	Category	Frequency	Percentage
Gender	Male	99	47.6
	Female	109	52.4
Year of study	3^rd^ year	105	50.5
	4^th^ year	103	49.5
Type of the nursing ward of the last clinical placement	Surgical	42	202
	Gynecology	22	10.6
	Medical	93	44.7
	Pediatrics	31	14.9
	Psychiatric	20	9.6
Type of the hospital in which clinical placement was held	Primary hospital	43	20.7
	General hospital	50	24.0
	Specialized care center	115	55.3
How many times did you meet NT during the latest clinical placement	Never	11	5.3
	1–2 times	69	33.2
	3 times	81	38.9
	Often	47	22.6

*n*: sample size, NT: nurse teachers.

**Table 2:** Level of satisfaction towards CLE among study participants.

Domains	Not satisfied		Satisfied	
	Frequency	Percentage (%)	Frequency	Percentage (%)
Content of the supervisory relationship	133	63.9	75	36.1
Role of the nurse teacher	136	65.4	72	34.6
Pedagogical atmosphere	104	50.0	104	50.0
Nursing care of the ward	135	64.9	73	35.1
Leadership style of the ward manager	124	59.6	84	40.4

**Table 3:** Multivariable logistic regression results showing factors associated with the satisfaction level of nursing students towards CLE, Southern Ethiopia, 2021 (*n* = 208).

Characteristics	Satisfaction towards CLE		COR (95%CI)	AOR (95%CI)
	Satisfied (%)	Not satisfied (%)		
**Gender**				
Male	14 (6.7%)	85 (40.9%)	1	1
Female	69 (33.2%)	40 (19.2%)	**10.473 (5.271, 20.808)**	**16.053 (6.397, 40.286)**
**Year of study**				
3^rd^ year	33 (15.9%)	72 (34.6%)	1	1
4^th^ year	50 (24.0%)	53 (25.2%)	**2.058 (1.170, 3.621)**	**6.296 (2.679, 14.796)**
**Type of the hospital**				
Primary hospital	32 (15.5%)	11 (5.3%)	**7.883 (3.544, 17.531)**	**2.961 (1.122, 7.815)**
General hospital	20 (9.6%)	30 (14.4)	1.806 (.897, 3.637)	1.04 (.46, 2.354)
Specialized care center	31 (14.9%)	84 (40.4)	1	1

Significant at *p* < 0.05; COR: result from bivariate and AOR: multivariable logistic regression. The bold values show significant associations between dependent and independent factors.

## Data Availability

Data sets used in this study are available from the corresponding author upon reasonable request.

## Abbreviations

CLE: Clinical learning environment
CI: Confidence interval
COR: Crude odds ratio
AOR: Adjusted odds ratio.
